# Comprehensive mapping of the human papillomavirus (HPV) DNA integration sites in cervical carcinomas by HPV capture technology

**DOI:** 10.18632/oncotarget.6809

**Published:** 2015-12-31

**Authors:** Ying Liu, Zheming Lu, Ruiping Xu, Yang Ke

**Affiliations:** ^1^ Key Laboratory of Carcinogenesis and Translational Research (Ministry of Education), Laboratory of Genetics, Peking University Cancer Hospital and Institute, Beijing, China; ^2^ Anyang Cancer Hospital, Henan Province, China

**Keywords:** HPV integration, cervical carcinoma, HPV capture, next generation sequencing

## Abstract

Integration of human papillomavirus (HPV) DNA into the host genome can be a driver mutation in cervical carcinoma. Identification of HPV integration at base resolution has been a longstanding technical challenge, largely due to sensitivity masking by HPV in episomes or concatenated forms. The aim was to enhance the understanding of the precise localization of HPV integration sites using an innovative strategy. Using HPV capture technology combined with next generation sequencing, HPV prevalence and the exact integration sites of the HPV DNA in 47 primary cervical cancer samples and 2 cell lines were investigated. A total of 117 unique HPV integration sites were identified, including HPV16 (*n* = 101), HPV18 (*n* = 7), and HPV58 (*n* = 9). We observed that the HPV16 integration sites were broadly located across the whole viral genome. In addition, either single or multiple integration events could occur frequently for HPV16, ranging from 1 to 19 per sample. The viral integration sites were distributed across almost all the chromosomes, except chromosome 22. All the cervical cancer cases harboring more than four HPV16 integration sites showed clinical diagnosis of stage III carcinoma. A significant enrichment of overlapping nucleotides shared between the human genome and HPV genome at integration breakpoints was observed, indicating that it may play an important role in the HPV integration process. The results expand on knowledge from previous findings on HPV16 and HPV18 integration sites and allow a better understanding of the molecular basis of the pathogenesis of cervical carcinoma.

## INTRODUCTION

Cervical carcinoma is one of the most commonly occurring cancers in women worldwide [1, 2], while greater than 99% of the disease contains human papillomavirus (HPV) sequences [3, 4]. Viral oncoproteins E6 and E7 encoded by HPV, which would respectively inactivate p53 and members of the pRb family, interfere with the cellular control mechanisms of the cell cycle. In addition, some studies demonstrated that these two oncoproteins also cooperatively disturb the mechanisms of chromosome duplication and segregation during mitosis and induce thereby severe chromosomal instability [5].

Studies have shown a significant correlation between integration and progression of cervical dysplastic lesions to invasive carcinomas. In pre-invasive lesions, HPV16 integration has already been found to occur in cervical intraepithelial neoplasia (CIN) [6], whereas in most cases of invasive carcinoma, HPV DNA sequences are frequently integrated into the host genome [7–10]. Although not much is known about the exact mechanisms of HPV genome integration, it is widely accepted that HPV integration can be a driver mutation in cervical carcinogenesis, with consequences influencing both the cellular and viral genomes. Recently, Akagi et al. presented a model of “looping” by which HPV integrant-mediated DNA replication and recombination may result in viral-host DNA concatemers, disrupting genes involved in oncogenesis and/or amplifying HPV oncogenes E6 and E7. Their results suggested that HPV directly promotes genomic instability [11]. Therefore, viral genome integration represents a crucial step in tumorigenesis, and elucidation of these particular integration events is pertinent for understanding HPV induced carcinogenesis.

Determining the DNA sequence of viral-cellular junctions would therefore indicate most directly HPV integration, as attempted in a number of previous studies where genomic HPV integration sites had been analyzed via many different assays. However, the precise identification of tiny stretches against a background of massive amount of episomal forms is an ongoing technical challenge. In addition, due to the nature of integration, which may be incorporated either as a single or multiple numbers of concatenated form, the latter can actually hinder integration detection [12]. Thus, development of more efficient methods would facilitate a comprehensive mapping of HPV integration sites to gain deeper insight into cervical carcinogenesis.

Here, in order to contribute to the understanding of the presence and the precise localization of HPV integration sites in cervical cancer, we studied a large series of cases using base-resolution genome-wide HPV capture technology with the next generation sequencing. More specifically, we simultaneously evaluate HPV prevalence and detailed characterization of the exact integration sites of the HPV DNA into the human genome in 47 primary cervical cancer samples and 2 cell lines.

## RESULTS

In this study, 49 DNA samples including 47 from fresh-frozen cervical carcinomas and 2 cervical cancer cell lines (SiHa and HeLa) were detected using the method of HPV capture combined with next generation sequencing (Supplementary Table S1).

### Distribution of HPV subtypes

All the DNA samples were captured with a total of 17 HPV types (6, 11, 16, 18, 31, 33, 35, 39, 45, 52, 56, 58, 59, 66, 68, 69, and 82). Of the 47 cancer samples, we were able to capture HPV DNA in 39 samples. Of these 39 samples, 30 were positive for HPV16. In addition to HPV 16, we also detected HPV18 (*n* = 4), 58 (*n* = 5), 45 (*n* = 1), 31 (*n* = 1). Two samples in this analysis harbored both types of HPV, HPV16 and HPV18 in T35, HPV18 and HPV58 in T9, respectively. However, in T35, the sequencing depth of HPV16 is 34-fold greater than that of HPV18, and in T9, the sequencing depth of HPV18 is 13 fold greater than that of HPV58. Therefore, only HPV16 in T35 and HPV18 in T9 were analyzed in subsequent HPV assay. SiHa was positive for HPV16, and HeLa was positive for HPV18 (Supplementary Table S1).

### Determination of potential HPV integration sites

**Figure 1:**
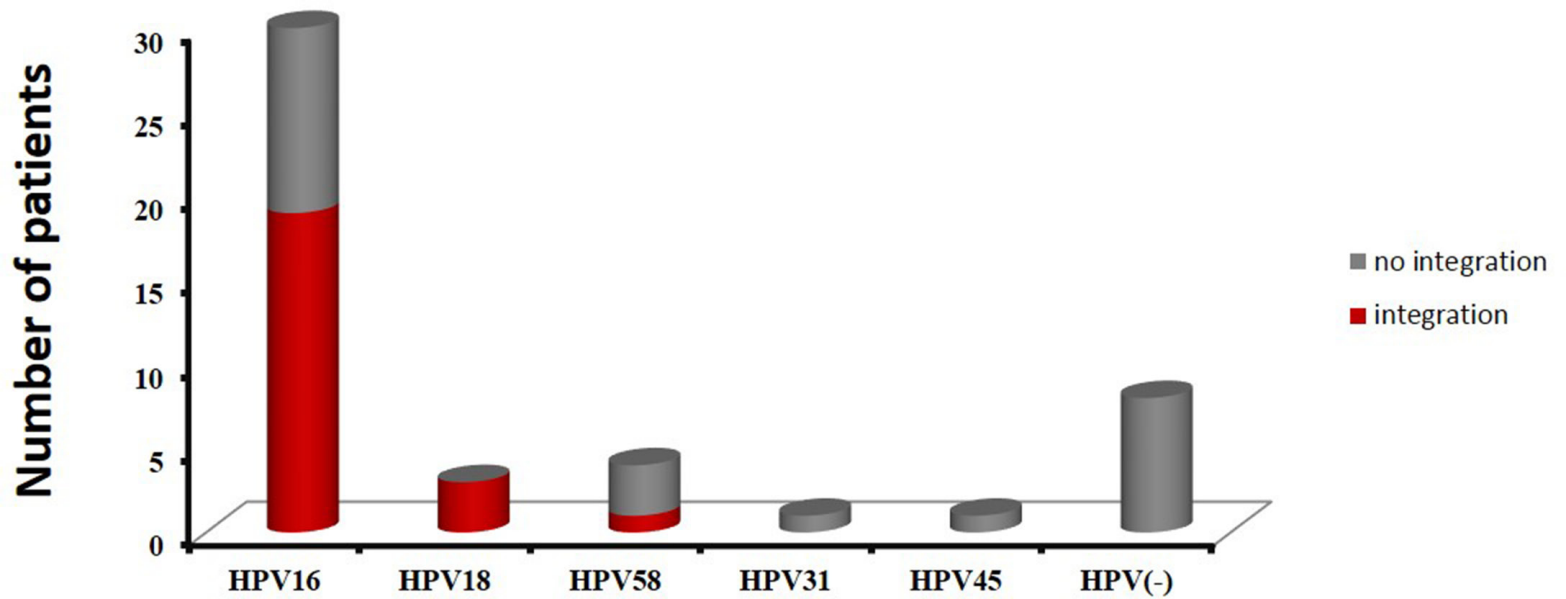
The distribution of HPV subtypes in 47 cervical cancer cases Red indicates samples with integration sites, and gray indicates samples with no integration.

As described in the Bioinformatics Analysis method, if a specific position has one or more discordant read pairs mapped with one end to a human chromosome and the other to the HPV reference genome, it will be considered as a potential HPV integration site. A total of 319 potential HPV integration sites were discovered in 25 HPV DNA-positive cases including 19 cases for HPV16, 3 cases for HPV18, 1 cases for HPV58, and 2 cell lines (SiHa, HeLa), with frequencies ranging from 1 to 59 per sample (Supplementary Table S1). The prevalence of integrated HPV16 DNA was 63% (19/30), while the prevalence of integrated HPV18 DNA reached 100% (3/3). Additionally, HPV integration was detected in one HPV58 DNA-positive sample, but the HPV45 and HPV31-positive cases showed no integration (Figure 1 and Supplementary Table S1).

### Validation and assay of HPV integration sites

In the attempt to confirm the newly uncovered HPV integration sites and further identify the exact integration site and the sequence between viral and cellular genome, all the 319 potential HPV integration sites were detected via targeted PCR amplification and Sanger sequencing. The Sanger sequencing validation rate based on one, two, three, or more than three discordant paired-end reads, was 3.7% (6/162), 47.8% (22/46), 44.4% (4/9), and 83.3% (85/102), respectively (Supplementary Table S1). The sequences of the fusion genes were first characterized by the NCBI human mega Blast database alignment tool and the UCSC Blat database to obtain the nucleotide resolution of the gene. A total of 117 unique HPV integration sites were identified, including HPV16 (*n* = 101), HPV18 (*n* = 7), and HPV58 (*n* = 9) (Table 1 and Supplementary Tables S1 and S2); thus providing further basis upon which to perform additional research on these validated integration sites.

**Table 1:** HPV-cellular DNA junctions confirmed by PCR amplification and Sanger sequencing

Sample name	#reads	HPV		Cellular sequence	
		subtype	breakpoint (ORF)^a)^	breakpoint^b)^	map
T4	4	HPV16	2046 (E1)	54417043	19q13.42
T4	5	HPV16	5190 (L2)	54417038	19q13.42
T8	2	HPV16	704 (E7)	59171259	17q23.2
T15	9	HPV16	3781 (E2)	73996849	13q22.1
T16	70	HPV16	2849 (E2)	17799430	7p21.1
T17	19	HPV16	2600 (E1)	53765119	19q13.42
T17	4	HPV16	3575 (E2/E4)	240309623	2q37.3
T17	1	HPV16	4448 (L2)	240281037	2q37.3
T18	78	HPV16	1063 (E1)	74594785	4q13.3
T20	831	HPV16	2689 (E1)	123715736	10q26.13
T20	400	HPV16	2863 (E2)	1019766	10p15.3
T23	8	HPV16	850 (E7)	32515477	20q11.22
T23	5	HPV16	884 (E1)	32487936	20q11.22
T23	14	HPV16	1277 (E1)	50054	18p11.32
T23	46	HPV16	2623 (E1)	8814272	6p24.3
T23	8	HPV16	2724 (E1)	30948621	20q11.21
T23	940	HPV16	2804 (E1/E2)	32516985	20q11.22
T23	12	HPV16	2891 (E2)	12645768	6p24.1
T23	21	HPV16	3163 (E2)	20462931	xp22.12
T23	8	HPV16	3182 (E2)	32516400	20q11.22
T23	131	HPV16	3328 (E2)	7328094	6p24.3
T23	43	HPV16	3344 (E2/E4)	7327990	6p24.3
T23	4	HPV16	3883 (E5)	156990726	3q25.31
T23	2	HPV16	4393 (L2)	17224129	12p12.3
T23	2	HPV16	4405 (L2)	99438640	2q11.2
T23	4	HPV16	4453 (L2)	32472391	20q11.22
T23	22	HPV16	4681 (L2)	7326060	6p24.3
T23	43	HPV16	5535 (L2)	20464412	xp22.12
T23	2	HPV16	5745 (L1)	103893590	11q22.3
T23	12	HPV16	6618 (L1)	32506420	20q11.22
T25	5	HPV16	1161 (E1)	31689429	10p11.22
T32	23	HPV16	3611 (E2/E4)	873456	9p24.3
T33	8	HPV16	958 (E1)	107223556	7q22.3
T33	4	HPV16	1558 (E1)	99425642	15q26.3
T33	2	HPV16	2744 (E1)	67500034	13q21.32
T33	8	HPV16	4911 (L2)	10702626	21p11.2
T34	5	HPV16	7 (LCR)	9283840	11p15.4
T34	2	HPV16	38 (LCR)	126566946	Xq25
T34	2	HPV16	1043 (E1)	9292546	11p15.4
T34	2	HPV16	1079 (E1)	128770421	8q24.21
T34	35	HPV16	1099 (E1)	128840275	8q24.21
T34	2	HPV16	1844 (E1)	25871313	5p14.1
T34	9	HPV16	2104 (E1)	128717682	8q24.21
T34	6	HPV16	2672 (E1)	64430179	4q13.1
T34	5	HPV16	2810 (E1/E2)	128578637	8q24.21
T34	4	HPV16	3355 (E2/E4)	126566939	xq25
T34	11	HPV16	3644 (E2)	128596370	8q24.21
T34	5	HPV16	3742 (E2)	9697576	11p15.4
T34	2	HPV16	5599 (L2/L1)	25871329	5p14.1
T34	3	HPV16	5629 (L2/L1)	128827759	8q24.21
T34	2	HPV16	5761 (L1)	9331203	11p15.4
T34	1	HPV16	7877 (LCR)	128579990	8q24.21
T35	4	HPV16	379 (E6)	129056613	8q24.21
T35	6	HPV16	738 (E7)	129040142	8q24.21
T35	2	HPV16	1362 (E1)	1806549	4p16.3
T35	3	HPV16	2941 (E2)	23578398	6p22.3
T35	29	HPV16	4963 (L2)	59909510	2p16.1
T35	107	HPV16	5609 (L2/L1)	154556784	7q36.2
T35	3241	HPV16	5619 (L2/L1)	129019228	8q24.21
T35	4	HPV16	7567 (LCR)	31618653	1p35.2
T36	4	HPV16	164 (E6)	22388589	11p14.3
T36	4	HPV16	2919 (E2)	85639302	2p11.2
T36	2	HPV16	3717 (E2)	85639289	2p11.2
T38	122	HPV16	384 (E6)	14288872	9p22.3
T38	668	HPV16	551 (E6)	14275445	9p22.3
T38	1	HPV16	858 (E7)	15070638	20p12.1
T38	3	HPV16	1690 (E1)	14330266	9p22.3
T38	1	HPV16	1975 (E1)	14869191	20p12.1
T38	2	HPV16	1987 (E1)	14872325	20p12.1
T38	5	HPV16	2508 (E1)	15079958	20p12.1
T38	9	HPV16	3647 (E2)	14246496	9p22.3
T38	3	HPV16	4652 (L2)	14388662	9p22.3
T38	1	HPV16	7480 (LCR)	15074857	20p12.1
T38	1394	HPV16	7708 (LCR)	14875592	20p12.1
T38	5	HPV16	7856 (LCR)	14236572	9p22.3
T39	19	HPV16	1661 (E1)	189584107	3q28
T39	22	HPV16	5300 (L2)	189584098	3q28
T42	1	HPV16	697 (E7)	163224769	1q23.3
T42	2	HPV16	1356 (E1)	96471972	Xq21.33
T42	4	HPV16	1728 (E1)	93013206	6q15
T42	6	HPV16	1942 (E1)	150420961	xq28
T42	151	HPV16	2059 (E1)	94561088	6q16.1
T42	2	HPV16	2189 (E1)	147585823	7q35
T42	4	HPV16	2370 (E1)	117973872	7q31.31
T42	2	HPV16	2816 (E2)	103005957	1p21.1
T42	499	HPV16	2991 (E2)	188223631	2q32.1
T42	4	HPV16	3331 (E2)	31137921	5p13.3
T42	2	HPV16	3402 (E2/E4)	42298926	2p21
T42	7	HPV16	4279 (L2)	117974122	7q31.31
T42	12	HPV16	4879 (L2)	231434760	2q37.1
T42	13	HPV16	5045 (L2)	86378287	14q31.3
T43	4	HPV16	777 (E7)	164142942	2q24.3
T43	36	HPV16	975 (E1)	73868139	13q22.1
T43	2	HPV16	3721 (E2)	45180348	2p21
T43	30	HPV16	4411 (L2)	73869942	13q22.1
T47	2	HPV16	340 (E6)	10902225	19p13.2
T47	4	HPV16	3501 (E2/E4)	62731602	17q24.1
T47	2	HPV16	3800 (E2)	31923670	6p21.33
T47	158	HPV16	3817 (E2)	57846210	6p11.2
SiHa	201	HPV16	3132 (E2)	74087562	13q22.1
SiHa	86	HPV16	3384 (E2/E4)	73788866	13q22.1
T9	294	HPV18	3782 (E2)	57925564	17q23.1
T31	6	HPV18	2918 (E2)	2539287	16p13.3
T44	45	HPV18	2233 (E1)	49741244	8q11.21
HeLa	496	HPV18	2497 (E1)	128241548	8q24.21
HeLa	702	HPV18	3100 (E2)	128233367	8q24.21
HeLa	5447	HPV18	5736 (L1)	128230632	8q24.21
HeLa	211	HPV18	7857 (LCR)	128234255	8q24.21
T11	29	HPV58	135 (E6)	45758413	18q21.1
T11	129	HPV58	661 (E7)	66426391	11q13.2
T11	4	HPV58	1244 (E1)	53599605	20q13.2
T11	14	HPV58	2164 (E1)	64506111	4q13.1
T11	2	HPV58	2195 (E1)	53582110	20q13.2
T11	59	HPV58	3791 (E2)	47684812	15q21.1
T11	1259	HPV58	5989 (L1)	53590867	20q13.2
T11	461	HPV58	6833 (L1)	53615729	20q13.2
T11	127	HPV58	7341 (LCR)	64506104	4q13.1

a) nucleotide position of viral-cellular breakpoints on the HPV genome (Alignment to NC_001526.2 for HPV16, NC_001357.1 for HPV18, and D90400.1 for HPV58);

b) Cellular nucleotide position of viral-cellular breakpoints on the human genome (Alignment to Hg19 human reference genome)

Abbreviations: T, Tumor; ORF, Open Reading Frame

SiHa and HeLa are cell lines with defined integration sites, which provide an invaluable standard for validation of diagnostic assays (Supplementary Figure S1 and Supplementary Figure S2). Consistent with other studies, two integration sites were observed in SiHa cell line at loci 13q22.1 [11, 13, 14] and four integration sites were observed in concatenated form in HeLa cell line at loci 8q24.21 [14–17].

### Mapping viral-cellular breakpoints to viral genome

**Figure 2:**
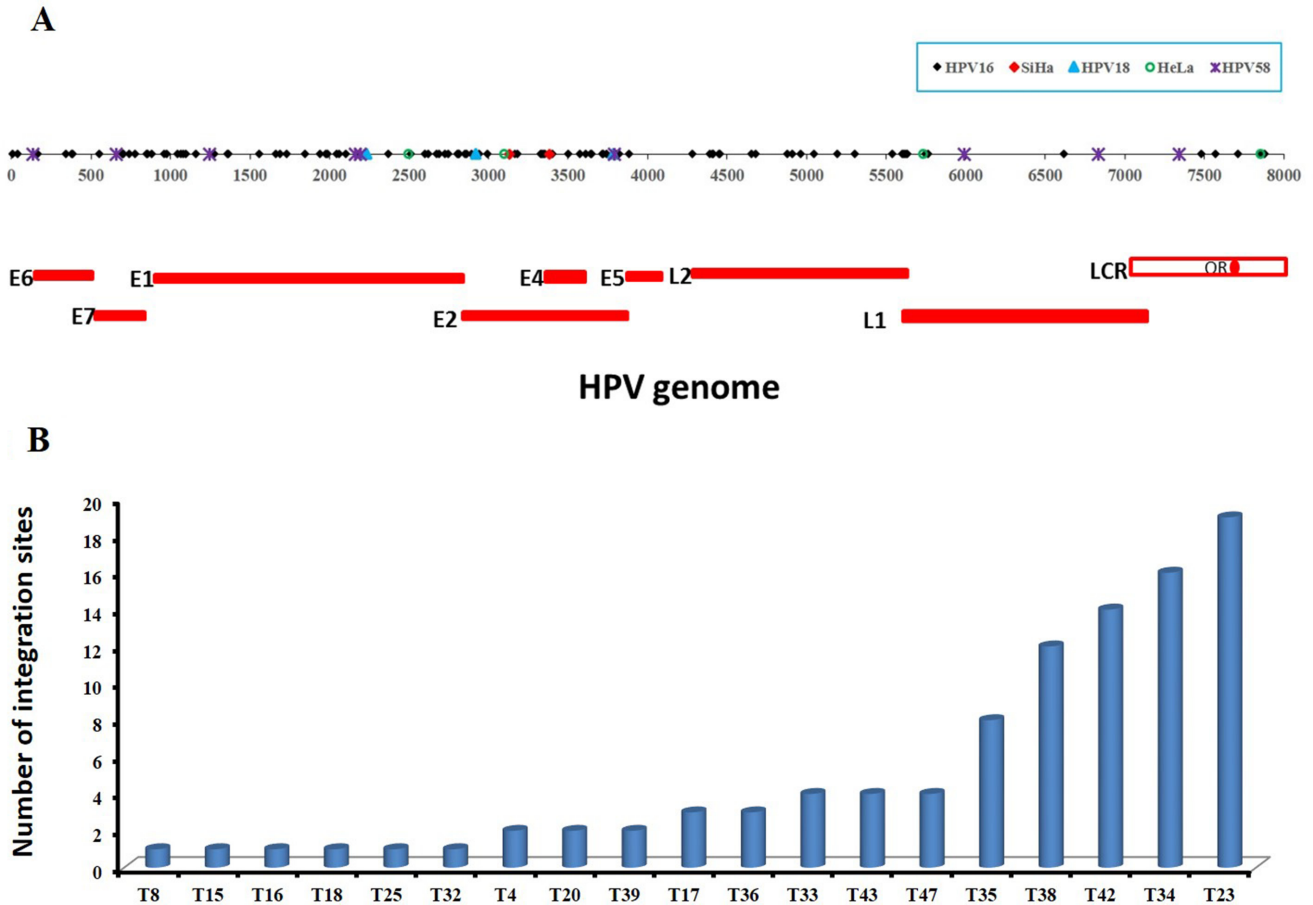
The distribution of validated integration breakpoints (**A**) 117 validated integration breakpoints, identified in 25 HPV-positive cases including cell lines of SiHa and HeLa, are shown according to position in HPV genome. 99 validated HPV16 integration breakpoints identified in 19 primary cervical cancer samples are shown in black, 2 HPV16 integration breakpoints in SiHa cell line in red, 3 HPV18 integration breakpoints in 3 primary cervical cancer samples in blue, 4 HPV18 integration breakpoints in HeLa cell line in green, and 9 HPV58 integration breakpoints in 1 primary cervical cancer sample in purple. (**B**) The frequencies of 99 validated integration sites in 19 HPV16-positive primary cervical cancer samples are shown, ranging from 1 to 19 per sample. See also Table 1 and Supplementary Table S1.

The 101 validated HPV16 integration sites in 20 samples including SiHa were shown to be broadly distributed across the whole genome with a unique distribution profile, including E6 (*n* = 5), E7 (*n* = 6), E1 (*n* = 31), E1/E2 (*n* = 2), E2 (*n* = 20), E2/E4 (*n* = 7), E5 (*n* = 1), L2 (*n* = 15), L2/L1 (*n* = 4), L1 (*n* = 3), and LCR (*n* = 7) (Table 1 and Figure 2A). Due to the different length of each fragment, direct comparisons of integration site frequency per se among the fragments is not feasible. From the distribution of points within the scatter plot (Figure 2A), the most common regions of integration are E2, E4, and E7, followed by E1, L2, and E6, and the sparsest are in LCR, E5, and L1.

Additionally, the frequencies of HPV16 integration sites ranged from 1 to 19 per sample (Figure 2B). That is, a single HPV integration site was found in 6 cases; 3 cases found two sites and 2 cases found three. Three additional cases found four sites each, and there was one case each with eight, twelve, fourteen, sixteen and nineteen sites (*n* = 99 sites) (Figure 2B and Supplementary Table S1). Altogether, 68% (13/19) of the HPV16 DNA-positive carcinomas were found to harbor more than one integration sites per sample.

While the 7 validated HPV18 integration sites in 4 samples including HeLa cell line were only mapped in the E1 (*n* = 2), E2 (*n* = 3), L1 (*n* = 1) and LCR (*n* = 1) region of HPV genome (Table 1 and Figure 2A), the frequency of integration was one per sample except four in HeLa cell line (Table 1 and Figure 2A).

9 validated HPV58 integration sites in one sample were mapped in the E6 (*n* = 1), E7 (*n* = 1), E1 (*n* = 3), E2 (*n* = 1), L1 (*n* = 2), and LCR (*n* = 1) (Table 1 and Figure 2A).

### Mapping viral-cellular breakpoints to chromosomal bands

**Figure 3:**
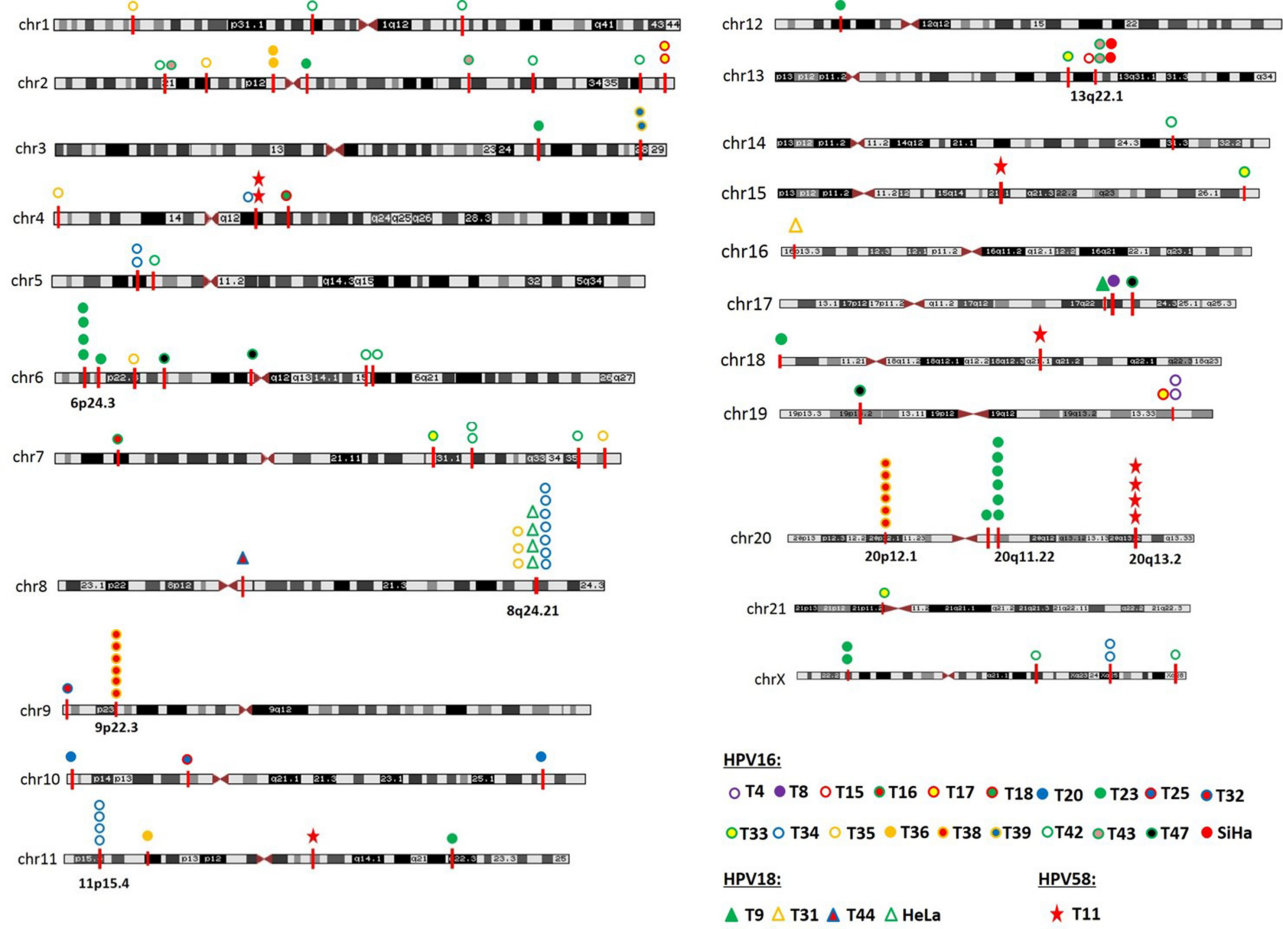
Chromosome localization of the 117 HPV integration sites in 25 cases The chromosomal reference of all viral-cellular breakpoints with respect to Giemsa-stained bands was taken from the UCSC database. 25 HPV-positive cases with integration sites are shown in unique color. HPV integration sites were mapped to almost all the human chromosomes, except chromosomes 22. HPV integration sites are clustered on chromosome 6p24.3, 8q24.21, 9p22.3, 11p15.4, 13q22.1, 20p12.1, 20q11.22, and 20q13.2.

Consistent with previous reports, integration sites were found to be located on virtually all the chromosomes, except chromosomes 22 (Figure 3). Besides, it is evident that several integration sites are clustered in some chromosomal regions, such as within the cytogenetic bands 6p24.3 (1.5M), 8q24.21 (0.8M), 9p22.3 (0.15M), 11p15.4 (0.4M), 13q22.1 (0.3M), 20p12.1 (0.2M), 20q11.22 (0.05M), and 20q13.2 (0.04M). Genomic distances among different viral integration sites in a “cluster”, defined by more than three unique HPV integration sites, spanned up to 1.5 Mb. We carefully analyzed the detailed characterization of these clusters, and divided them into three groups. In the first group, a cytogenetic band showed three or more HPV integration sites for each sample, and it is plausible that they are integrated in a concatenated array, consistent with the recently suggested looping model [11]. For example, HPV integrant clusters mapped to 6p24.3 in T23 (*n* = 4), 9p22.3 in T38 (*n* = 6), 11p15.4 in T34 (*n* = 4), 20p12.1 in T38 (*n* = 6), 20q11.22 in T23 (*n* = 6), and 20q13.2 in T11 (*n* = 4). In the second group, some integrant clusters arose from at least three individual cases, and they may represent authentic hot spots, such as 13q22.1 in SiHa (*n* = 2), T15 (*n* = 1), and T43 (*n* = 2). In the last group, the clusters showed both of the aforementioned properties, such as 8q24.21 in HeLa (*n* = 4), T34 (*n* = 7), and T35 (*n* = 3) (Figure 3).

### Characterization of genomic DNA sequences adjacent to HPV integration breakpoints

Through PCR amplification and Sanger sequencing, we obtained the viral-cellular junction sequences. There were three patterns of host-virus sequences after aligning to the reference viral genome and human genome (hg19), including overlapping, inserting some unaligned nucleotides, and direct ligation (Supplementary Table S2). Overlapping, defined by nucleotides shared between viral and cellular sequence, was the most prevalent pattern accounting for 67% of the 117 validated viral-cellular sequences. Since some nucleotides between viral and cellular sequence were not aligned to a particular genomic sequence, we categorized them as the insertion of some unaligned nucleotides, which group amounts to approximately 27% of all 117 sequences. About 6% of the 117 viral-cellular sequences were present with direct ligation. Additionally, information of the cellular sequences was observed and collected with RepeatMasker. It was found that 51.3% of the 117 integration sites were distributed in repeating elements throughout the human genome (Supplementary Table S2), consistent with the actual proportion (50%) of repetitive sequence in the human genome [18].

All integration loci were checked for the presence of fragile sites. Of the 117 integration loci identified, 69 (59%) were located in or close to a fragile site (Supplementary Table S2): 20 (17%) within common or rare fragile sites and 49 (42%) up to 5 Mb adjacent to fragile sites. 48 (41%) integration sites were not associated with fragile sites.

Meanwhile, the genomic regions within 50 kb of the viral-cellular junctions were investigated. Approximately 47% (55/117) of the 117 confirmed HPV integration sites were located within cellular genes, and 54 cellular DNA breakpoints were located in introns and one breakpoint was located in exon (Supplementary Table S2). Moreover, the following cellular genes occurred in at least two unique HPV integration sites, including CACNG7 (2), CAPG (2), HDAC4 (2), TP63 (2), CAGE1 (2), PVT1 (5), MACROD2 (6), and NFIB (6). The targeted genes are in the same orientation as the cellular sequence of viral-cellular junctions in 31 HPV integration sites, and in the opposite orientation in 24 HPV integration sites (Supplementary Table S2).

### Correlation between HPV status and clinical information

**Figure 4:**
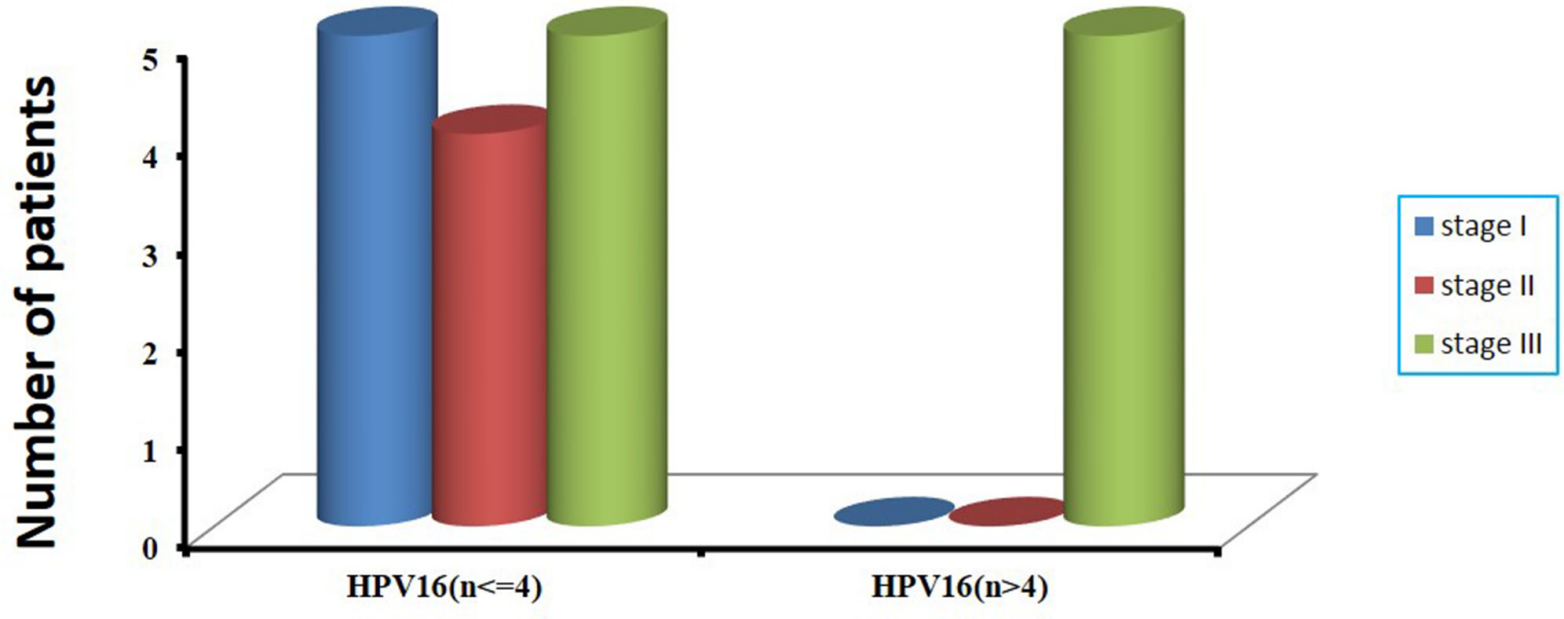
The correlation of number of integrated sites with clinical stages in 19 HPV16-positive cervical cancer samples According to the frequency of HPV16 integration sites per sample, we divide them into two groups, one is more than four HPV16 integration sites, and the other is no more than four HPV16 integration sites (*x*-axis). The number of patients with clinical stages in two groups is shown (*y*-axis).

Among the 47 patients with cervical cancer, 17 patients had received chemotherapy and radiotherapy before surgery, and the remaining 30 patients had not been treated before surgery (Supplementary Table S1). The prevalence of HPV DNA in 17 patients with treatment and in 30 patients without treatment before surgery was 76.5% (13/17), and 86.7% (26/30), respectively. However, there was no statistically significant difference (76.5% vs 86.7%, *P* = 0.435). In consideration of HPV integration status, we found that in 13 HPV DNA-positive patients treated with radiotherapy and chemotherapy before surgery, HPV integrations were not detected in 10 patients, including 8 patients positive for HPV16 and 1 patient positive for HPV45 and 1 patient positive for HPV58. Altogether, in 17 patients with cervical cancer who had received radiotherapy and chemotherapy before surgery, 82.4% (14/17) showed HPV negative or no HPV integration sites (Supplementary Table S3). According to the frequency of HPV16 integration sites per sample, we found that all the 5 cervical cancer cases harboring more than four HPV16 integration sites had received diagnosis of clinical stage III carcinoma (Supplementary Table S1 and Figure 4). No significant difference was found between HPV integration and age at diagnosis (Supplementary Table S3).

## DISCUSSION

Utilization of the PCR-based approach has prevailed in the existing attempts of detection of HPV integration sites in the human genome, such as Amplification of Papillomavirus Oncogene Transcripts (APOT) in discriminating HPV mRNAs derived from integrated and episomal viral genomes [19, 20], Restriction Site PCR (RS-PCR) [21, 22], Southern blot and Detection of Integrated Papillomavirus Sequences (DIPS) in detecting integrated HPV DNA regardless of its transcriptional status [15], and Real-time PCR, which can determine the physical status (integrated or episomal) of HPV estimated by calculating HPV E2:E6/E7 ratio by real-time PCR amplification of HPV E2 and E6/E7 [23]. However, all these assays usually are time-consuming and fall short of sensitivity, because of a battery of type-specific primers adopted and innate random distribution of HPV integration sites. Furthermore, sensitivity may be heavily influenced by a number of factors, such as the virus copy number, the physical status (episomal or integrated of HPV), and integrated form (in a single copy or in concatenated form) [12]. To overcome these and other potential limitations, HPV capture combined with next generation sequencing, which presents a more efficient method as described here, is not only able to detect the signals of multiple virus in a single run with high specificity and sensitivity, but has the ability to distinguish, from a diverse background of mainly episomal or concatenated forms, a discreet number of integrated forms, and further determinate the sequence between viral and cellular junctions.

HPV capture in this study was designed based on probes from full-length HPV genome of 17 types. Thus, the HPV subtypes and HPV DNA integration sites were simultaneously detected in 49 cervical cancer samples (47 primary tumors and 2 cell lines). Out of which 82.9% of the 47 cervical cancer cases was HPV DNA positive, including HPV16(63.8%), HPV18(8.5%), HPV58(10.6%), HPV45 (2.1%), and HPV31 (2.1%). These percentages are relatively well aligned with existing findings [24–26], that is, HPV16 is by far the most prevalent type responsible for more than 50% of all the cervical cancer cases worldwide, followed by HPV18 and HPV58 and other high-risk HPV types that are less prevalent.

The sensitivity of the HPV capture combined with sequencing can be estimated from the results obtained from the well characterized cervical carcinoma cell lines SiHa and HeLa. SiHa, a HPV16-positive cervical cell line, harbors only one HPV16 DNA copy per cell [27, 28]. Two validated HPV integration sites were found in SiHa cell line, as the same sites reported previously [10–12] (Supplementary Figure S1 and Supplementary Table S2). Four HPV18 integration sites were detected on the chromosomal band 8q24.21 in HeLa cell line (Supplementary Figure S2 and Supplementary Table S2), which were consistent with previous studies [15, 16]. Thus, our analysis had achieved 100% sensitivity and specificity compared with previous reported sites for SiHa and HeLa [11, 15, 16].

With this strategy, 117 unique and validated HPV integration sites and high-quality nucleotide sequences of the viral-cellular junctions were obtained. Of the 30 HPV16 DNA-positive cervical cancer samples, 63% (19/30) harbored HPV integration sites, similar to previously reported percentages [10, 29], indicating the presence of a large number of integrated viral genomes in HPV16-positive cervical carcinomas. Moreover, 68% (13/19) of the HPV16 DNA-positive carcinomas were found to harbor more than one DNA junctions per sample. This high percentage of multiple HPV16 integration sites exceeds 15~20% reported in previous studies using DIPS- PCR and RS-PCR methods [22, 30, 31], and also exceeds 49% reported in a recent study using TEN16 (Tagging, Enrichment, and Next-generation sequencing of HPV16) assay [10], indicating the more effective performance of HPV capture combined with sequencing adopted in this study for HPV DNA integration site analysis.

It is worthy to note that our data, obtained from 101 validated HPV16 integration sites including 2 integration sites from SiHa cell line, indicated that all the regions of HPV genome can be targeted for the linkage to cellular sequences, even E6 and E7. Doubt arises on the feasibility of the widely used E2:E6/E7 ratio by real-time PCR amplification to determine the physical status of HPV infection, which is based on the assumption that the viral E2 gene is frequently disrupted upon HPV integration, and the HPV E6 and E7 open reading frames are almost always retained, whereas other portions of the HPV genome may be deleted [32]. In contrast, the breakpoints of HeLa and 3 other HPV18-positive cervical cancer cases observed to occur at viral E1, E2, L1 and LCR region have been reported [15, 33].

Of the 4 HPV18 DNA-positive cervical carcinomas including HeLa, 100% (4/4) harbored HPV integration sites. This percentage conforms to the estimation of 100% of HPV18-positive cervical carcinomas harboring integrated viral DNA by Southern analysis [33]. The high integration frequency of HPV18 may be related to its greater transforming efficiency *in vitro* and its reported clinical association with more aggressive cervical cancers [34].

PCR and Sanger sequencing were used to verify the HPV integration breakpoints based on one or more discordant paired-end reads. Although the low validation rate for one to three discordant paired-end reads may arise from technical error or integration occurred in a small subset of cancer cells, practically, the criterion using more than three discordant read pairs can cover most breakpoints identified (85/117). It should be noted that no explanation could sufficiently capture the failure of the remaining 16.7% of integration sites for validation based on more than three discordant reads, such as the number of discordant paired-end reads, and the proportion of repetitive sequence in the human genome. Additional research is warranted to reduce the false positive rate.

Viral integration within fragile sites has previously been reported in several studies [22, 35]. Fragile sites are genomic regions prone to chromosome breaks that facilitate foreign DNA integration. Of the 117 integration loci identified, 69 were located in or close to a fragile site. It is note that the clustered integration sites on 6p24.3, 8q24.21, 13q22.1, and 20p12.1 were located within 1.8, 1.8, 0.8, and 3 Mb, respectively, of an adjacent fragile site, and that the clusters on 9p22.3, 11p15.4, 20q11.22, and 20q13.2 were located >5 Mb distant to any known fragile site. Thus, it is tempting to speculate that unknown fragile sites may be identified based on the HPV integration hotspots.

In total, 55 (47%) of the 117 confirmed HPV integration sites were located within cellular genes, most frequently in intronic regions. Moreover, several cellular genes existed in two or more unique HPV integration sites in the current study, including CACNG7, CAPG, HDAC4, TP63, CAGE1, PVT1, MACROD2, and NFIB. HPV integration into an intron will probably disturb expression of the targeted cellular gene. However, only few examples exist, because a direct link between HPV integration and gene alteration must be verified by functional data. TP63, a member of the p53 protein family, acts as a tumor suppressor protein and aberrant expression was noted for several cancer entities including cervical cancer [36, 37], supporting the assumption that insertional mutagenesis of cellular genes by HPV integration contributes to cervical carcinogenesis.

In our data, overlapping was the most prevalent pattern accounting for 67% of the 117 validated viral-cellular sequences. A significant enrichment of overlapping nucleotides shared between the human genome and HPV genome at integration breakpoints was observed, indicating that it may play a vital role in the HPV integration process.

Additionally, we observed that the prevalence of HPV DNA in 17 patients who had been treated with radiotherapy and chemotherapy before surgery was lower than that in the group of 30 patients without the pre-operative procedures, but no statistically significant difference was found. While considering the status of HPV integration, we found that 82.4% showed negative for HPV or an absence of HPV integration sites in 17 patients with cervical cancer who had received pre-operative radiotherapy and chemotherapy. These results might indicate that radiotherapy and chemotherapy before surgery had some impact on HPV in cervical carcinoma, especially on HPV integration. A plausible explanation for the decrease of HPV integration is that these cells have been more effectively cleared by treatment. Additionally, we found that all the cervical cancer cases harboring more than four HPV16 integration sites showed clinical features and diagnosis of stage III carcinoma, indicating that the complexity of HPV integration might be associated with advanced cervical cancers.

Taken together, the method of HPV capture combined with sequencing allow a better analysis of multiple HPV subtypes at the same time and to identify the precise nucleotide sequences at the viral-cellular junctions as well as the affected cellular genes. The high number of 117 validated unique HPV integration sites deduced in the study demonstrates the effective performance of this strategy at single-nucleotide resolution. HPV integration is a special pattern of cancer mutation, which has the well-established potential to affect the cellular genes involved in cancer carcinogenesis. Therefore, identification of HPV integration in cervical cancers can provide new insight into the understanding of the cancer genome projects and its role in the pathogenesis of cervical carcinoma. Furthermore, because of the intrinsic random distribution of HPV integration sites, the efficient determination of HPV integration sites would allow them to be served as individualized markers in cervical cancer screening and in the follow-up of patients for the early detection of recurrent disease and metastasis in the future.

## MATERIALS AND METHODS

### Sample collection and cell lines

A total of 47 fresh tissue specimens were collected from patients with cervical cancers who had undergone surgeries at Anyang Cancer Hospital, Henan province, China, between 2009 and 2010. Median age of these patients was 51 years with range 29 to 81 years. Among whom 42 had squamous cell carcinoma, 3 had adenocarcinoma and 2 adenosquamous carcinoma. The HPV16- and HPV18-positive human cervical carcinoma cell lines SiHa and HeLa with defined integration sites served as positive control. SiHa and HeLa cell lines had been obtained from AACT many years ago, and were often used for research purposes. Before undergoing experimentation in this study, these two cell lines had been identified using a PCR-based short tandem repeat (STR) genotyping method.

Individual informed consents had been collected from all study participants. This study received ethical approval from the Institutional Review Board of the hospital.

### DNA preparation

DNA was extracted using DNeasy Blood & Tissue kit (Qiagen, Hilden, Germany) according to the manufacturer's protocol. The DNA concentration was determined via a Nano-Drop (NanoDrop Technologies, Wilmington, Delaware USA).

### Overall process of HPV capture and sequencing

#### Prepare DNA whole-genomic library

In order to establish the whole genomic library with the targeted gene specifically, a stretch of 3~5 μg genomic DNA was sheared to about 150 bp fragments by Covaris S2. These fragments were subsequently end blunted, “A” tailed, adaptor ligated and amplified 10 cycles by PCR. Out of which 1 μl of the prepared library samples was quantified using Nanodrop 2000, and 3 μl identified in 2% agarose gel and the final size of the electrophoresis fragment was around 300bp~500 bp.

### Capture targeted gene regions and sequencing

HPV probes were designed according to full- length genome of 17 HPV types (6, 11, 16, 18, 31, 33, 35, 39, 45, 52, 56, 58, 59, 66, 68, 69, and 82) by MyGenostics (MyGenostics, Baltimore, MD, USA). The overall experiment was conducted according to the manufacturer's protocol. In brief, the whole-genomic libraries were hybridized with HPV probes (MyGenostics GenCap Technology), adsorbed onto the beads via biotin and streptavidin magnetic beads, and the uncaptured DNA fragments were removed by washing. Then the eluted fragments containing the targeted gene were enriched by 18 cycles of PCR to generate libraries for sequencing. Libraries were quantified and sequenced for paired-end 100bp using the Illumina HiSeq 2000 sequencer (Illumina Inc., San Diego, CA, USA).

### Bioinformatics analysis method

The 100 bp paired-end reads were preceded into bioinformatics analysis. For quality control, we first filtered low quality reads using the Trim Galore program. Then, 3′/5′ adaptors were trimmed using the Cutadapt program implemented in Trim Galore, thereby rendering high quality clean reads, whose quality value exceeds 20 and read length greater than 80 bp. The high quality reads were obtained for subsequent analysis.

Illumina clean reads were mapped to human genome (GRCh37/hg19) and HPV genome of 17 types (6, 11, 16, 18, 31, 33, 35, 39, 45, 52, 56, 58, 59, 66, 68, 69, and 82) using the BWA program. The quality scores were recalibrated and realigned to reference sequences using the GATK software package. For the potential HPV integration sites in the human genome, we aligned the Illumina clean reads to the reference human genome hg19 using the BWA program, and simultaneously performing recalibration and realignment with GATK. The paired-end read, uniquely mapped with one end to a human chromosome and the other to the HPV reference genome, is identified as a discordant read pair. If a specific position has one or more discordant read pairs, it would be considered as a potential HPV integration site and the breakpoints of which were then identified using the BreakDancer program. The integration sites were annotated according to human genome (GRCh37/hg19) from the UCSC database and HPV genome.

The sample was considered HPV DNA positive when the average sequencing depth was more than 10 and more than 50% of the virus genome's total length was covered by at least 4x.

The average sequencing depth was calculated based on sequencing tags containing HPV sequences and according to the following formula: Sequencing Depth = Total HPV bases/genome size. Genome size here means the size of the specific HPV genome.

### PCR amplification and Sanger sequencing

To validate the potential HPV integration sites, we designed primers to amplify regions of interest by PCR. PCR products were sequenced directly from both directions using conventional Sanger sequencing. If unsuccessful, the integration sites were identified by TA cloning of PCR products and sequencing. All sequences of the fusion genes were characterized by the NCBI human mega Blast database alignment tool and the UCSC Blat database.

### Statistical analysis

Fisher's exact test was used for statistical analysis in the present study. *P*-value of less than 0.05 was considered as statistically significant. All the *P*-values presented are two-sided.

## SUPPLEMENTARY MATERIALS TABLES AND FIGURES


