# Supplementary material for: Comprehensive mapping of the human papillomavirus (HPV) DNA integration sites in cervical carcinomas by HPV capture technology
Source: Oncotarget. 2015 Dec 31;7(5):5852–64. doi: 10.18632/oncotarget.6809 (PMC4868726; doi:10.18632/oncotarget.6809)
Supplement: Supplementary file 1 [file oncotarget-07-5852-s001.pdf]

Comprehensive mapping of the human papillomavirus (HPV) DNA integration sites in cervical carcinomas by HPV capture technology

Supplementary Materials

Supplementary Table S1: Summary of 49 cancer samples analyzed in this study: HPV viral status and clinical information

Sample name	Age (years)	HPV status	Sequencing depth	*N* of validated integration sites on the basis of different reads					Status of radiotherapy and chemotherapy	Clinical stage	Pathology
				Total	> 3	3	2	1			
T1	54	Negative							Yes	IIIb	squamous cell carcinoma
T2	43	Negative							Yes	IIb	squamous cell carcinoma
T6	40	Negative							No	Ib	squamous cell carcinoma
T7	51	Negative							No	IIa	squamous cell carcinoma
T12	55	Negative							Yes	IIIb	squamous cell carcinoma
T14	61	Negative							Yes	IIIb	adenocarcinoma
T27	68	Negative							No	IIb	squamous cell carcinoma
T46	32	Negative							No	Ia	squamous cell carcinoma
T3	41	HPV16	128						Yes	IIb	squamous cell carcinoma
T5	59	HPV16	15						Yes	Ia	squamous cell carcinoma
T10	59	HPV16	12						No	IIIb	adenocarcinoma
T21	51	HPV16	12						No	IIIb	adenocarcinoma
T26	54	HPV16	62						No	IIb	squamous cell carcinoma
T28	36	HPV16	150						Yes	IIb	squamous cell carcinoma
T29	45	HPV16	159						Yes	IIb	squamous cell carcinoma
T37	45	HPV16	52						Yes	Ia	squamous cell carcinoma
T40	55	HPV16	16						Yes	IIIb	squamous cell carcinoma
T41	44	HPV16	112						Yes	IIIb	squamous cell carcinoma
T45	52	HPV16	20						Yes	Ia	squamous cell carcinoma
T15	44	HPV16	338	1/1	1/1				No	Ib	adenosquamous carcinoma
T18	54	HPV16	454	1/1	1/1				No	IIb	squamous cell carcinoma
T25	36	HPV16	271	1/1	1/1				No	IIIb	squamous cell carcinoma
T32	35	HPV16	157	1/1	1/1				No	IIIb	squamous cell carcinoma
T16	60	HPV16	834	1/3	1/2			0/1	No	IIIb	squamous cell carcinoma
T8	40	HPV16	15	1/5		0/1	1/3	0/1	No	IIa	squamous cell carcinoma
T20	41	HPV16	1511	2/2	2/2				No	Ia	squamous cell carcinoma
T39	57	HPV16	120	2/2	2/2				No	IIIb	squamous cell carcinoma
T4	48	HPV16	15	2/3	2/2			0/1	Yes	IIb	squamous cell carcinoma
T17	41	HPV16	11412	3/14	2/2		0/2	1/10	No	IIa	squamous cell carcinoma
T36	29	HPV16	1689	3/3	2/2		1/1		No	Ia	squamous cell carcinoma
T33	46	HPV16	6384	4/13	3/3		1/1	0/9	No	Ia	squamous cell carcinoma
T47	59	HPV16	10815	4/22	2/3		2/3	0/16	No	IIIb	squamous cell carcinoma
T43	47	HPV16	353	4/4	3/3		1/1		No	Ib	squamous cell carcinoma
T35[a)]	71	HPV16 and HPV18	5524 and 160	8/26	6/7	1/1	1/2	0/16	No	IIIb	squamous cell carcinoma
T38	62	HPV16	8377	12/38	6/8	2/2	1/3	3/25	No	IIIb	squamous cell carcinoma
T42	81	HPV16	6349	14/39	9/13	0/3	4/10	1/13	No	IIIb	squamous cell carcinoma
T34	81	HPV16	7731	16/39	8/12	1/1	6/13	1/13	No	IIIb	squamous cell carcinoma
T23	56	HPV16	10220	19/31	16/19	0/1	3/4	0/7	No	IIIb	squamous cell carcinoma
T31	48	HPV18	77	1/2	1/2				No	IIIb	squamous cell carcinoma
T44	47	HPV18	69	1/1	1/1				No	IIIb	squamous cell carcinoma

T9[b)]	39	HPV18 and HPV58	380 and 29	1/1	1/1				Yes	IIIb	adenosquamous carcinoma
T11	56	HPV58	2979	9/59	8/8		1/3	0/48	Yes	IIIb	squamous cell carcinoma
T19	52	HPV58	2676						No	IIb	squamous cell carcinoma
T24	33	HPV58	556						No	Ia	squamous cell carcinoma
T30	38	HPV58	11						Yes	IIb	squamous cell carcinoma
T22	53	HPV31	79						No	Ia	squamous cell carcinoma
T13	64	HPV45	60						Yes	IIIb	squamous cell carcinoma
SiHa		HPV16	228	2/2	2/2						
HeLa		HPV18	3259	4/6	4/4			0/2			

[a)]the sample of T35 harbors HPV16 and HPV18, and the sequencing depth of HPV16 is 34-fold greater than that of HPV18. So, only HPV16, a main type of HPV in sample of T35, is analyzed in subsequent HPV assay.

[b)]the sample of T9 harbors HPV18 and HPV58, and the sequencing depth of HPV18 is 13 fold greater than that of HPV58. So, only HPV18, a main type of HPV in sample of T9, is analyzed in subsequent HPV assay.

Supplementary Table S2: HPV-cellular DNA junctions confirmed by PCR amplification and Sanger sequencing

Supplementary Table S3: Correlation between HPV status and clinicopathological parameters

Clinical Characteristics	*N*	HPV DNA		*P*	HPV integration		*P*
		Negative (%)	Positive (%)		No (%)	Yes (%)	
**Age (year)**							
< 50	23	3 (13.0)	20 (87.0)	0.701	10 (43.5)	13 (56.5)	0.387
> 50	24	5 (20.8)	19 (79.2)		14 (58.3)	10 (41.7)	
**Stage**							
I + II	25	5 (20.0)	20 (80.0)	0.706	16 (64.0)	9 (36.0)	0.082
III	22	3 (13.6)	19 (86.4)		8 (36.4)	14 (63.6)	
**Preoperative radiotherapy and chemotherapy**							
No	30	4 (13.3)	26 (86.7)	0.435	10 (33.3)	20 (66.7)	**0.002**
Yes	17	4 (23.5)	13 (76.5)		14 (82.4)	3 (17.6)	

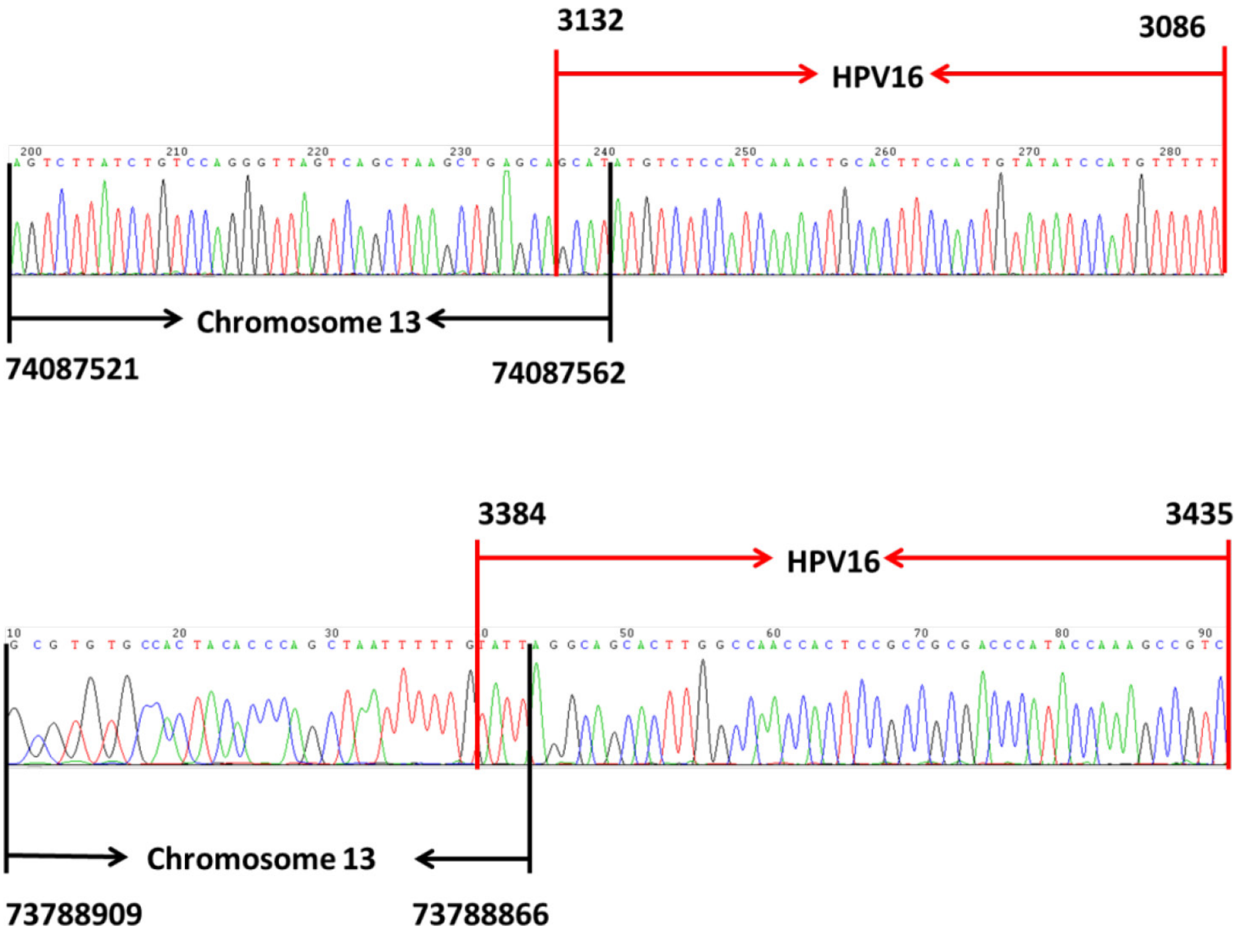

Supplementary Figure S1: Sequence analysis of two viral-cellular junctions found in SiHa cells that resulted from human papillomavirus (HPV) 16 integration into human chromosome 13. The sequence between two red lines belongs to HPV16, and the sequence between two black lines belongs to chromosome 13. Some nucleotides between red line (left) and black line (right) located in the middle are shared between both viral and cellular sequence. The number of sequences is annotated according to GenBank accession number NC_001526.2 for HPV16 and Hg19 human reference genome.

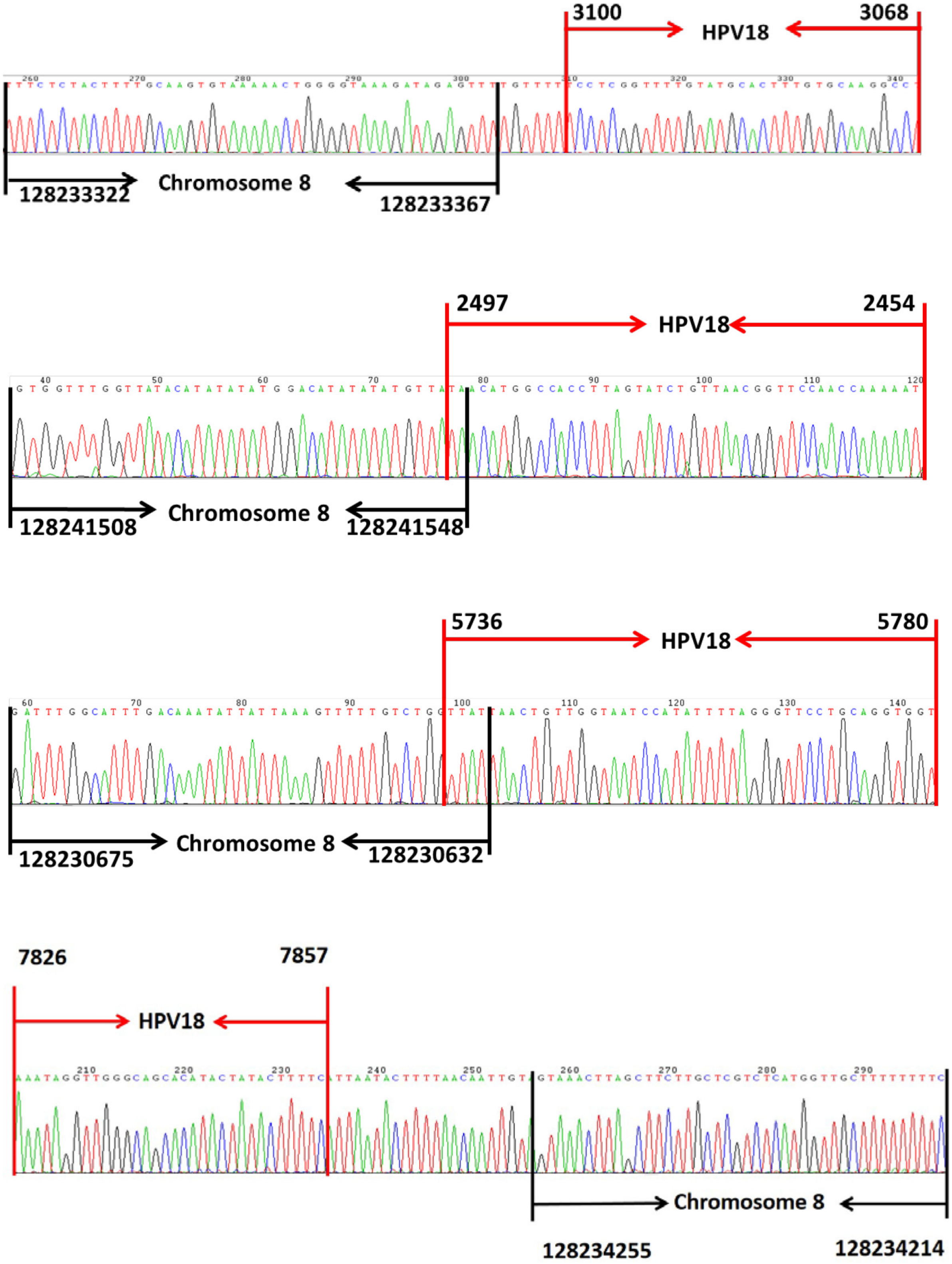

Supplementary Figure S2: Sequence analysis of four viral-cellular junctions found in HeLa cells that resulted from human papillomavirus (HPV) 18 integration into human chromosome 8. The sequence between two red lines belongs to HPV18, and the sequence between two black lines belongs to chromosome 8. There are two forms according to some nucleotides between red line and black line located in the middle. One is overlapping, defined by containing nucleotides shared between both viral and cellular sequence. The other is inserting some unaligned nucleotides, since these nucleotides were not assigned to a particular genomic sequence. The number of sequences is annotated according to GenBank accession number NC_001357.1 for HPV18 and Hg19 human reference genome.
